# Supplementary figures and images for: Single-cell sequencing analysis related to sphingolipid metabolism guides immunotherapy and prognosis of skin cutaneous melanoma
Source: Front Immunol. 2023 Nov 23;14:1304466. doi: 10.3389/fimmu.2023.1304466 (PMC10701528; doi:10.3389/fimmu.2023.1304466)

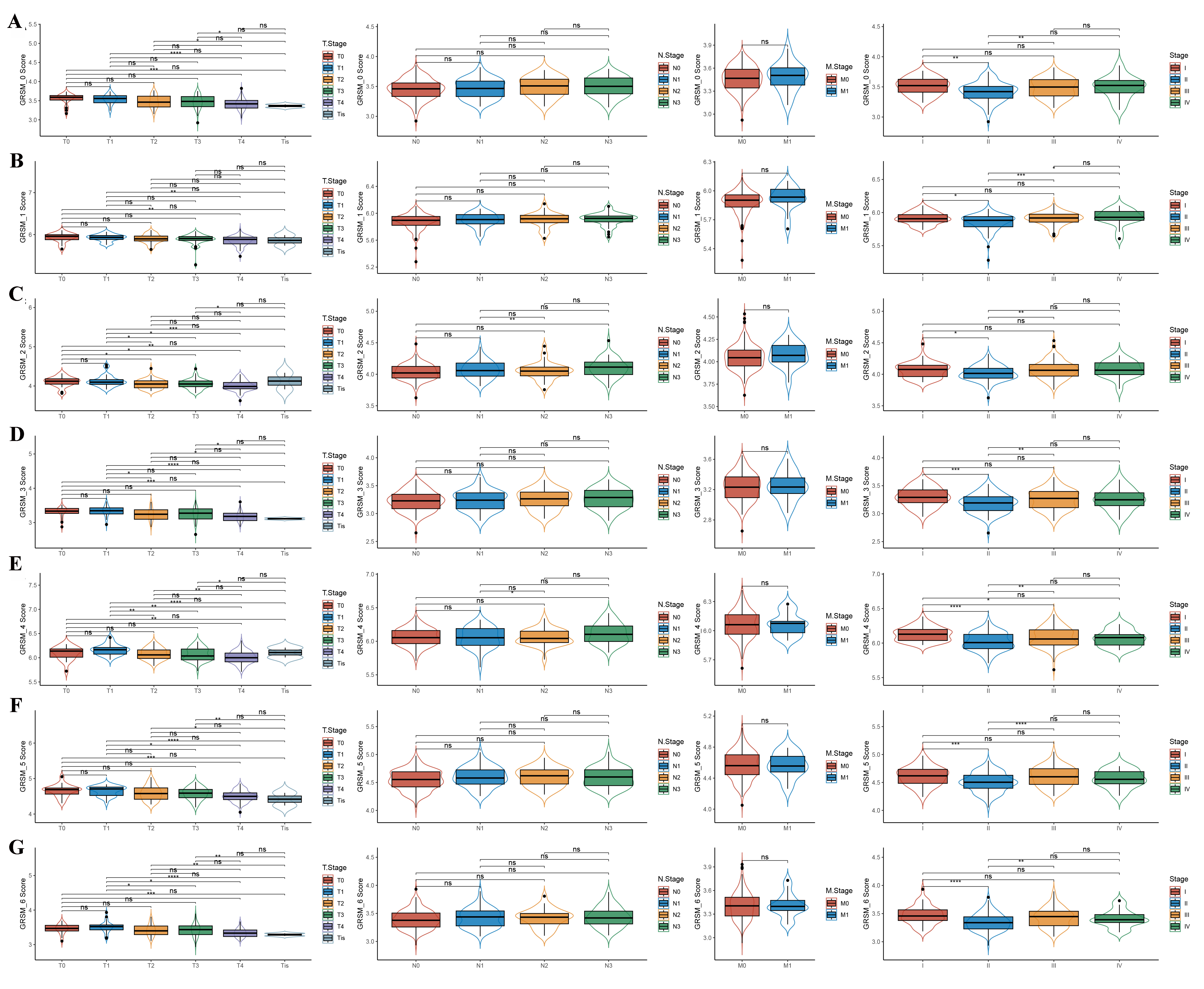

Supplement: Supplementary Figure 1 — (A-G) The correlation between SRGs clusters and clinical characteristics. [file Image_1.tif]

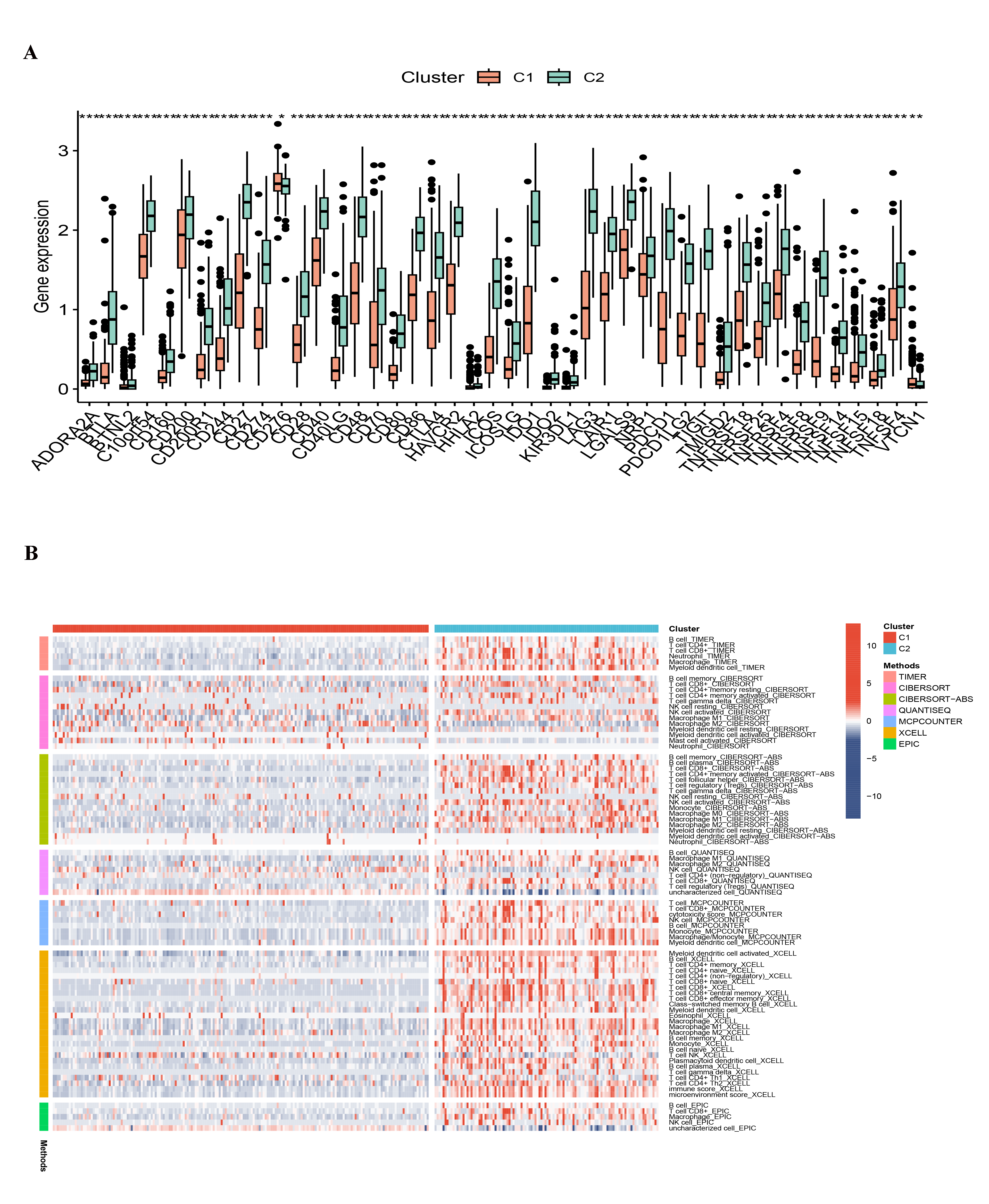

Supplement: Supplementary Figure 2 — The analysis of immune checkpoint inhibitors. (A) A significant association between the C2 cluster and the expression of most immune checkpoints. (B) The analysis of immune cell infiltration. [file Image_2.tif]

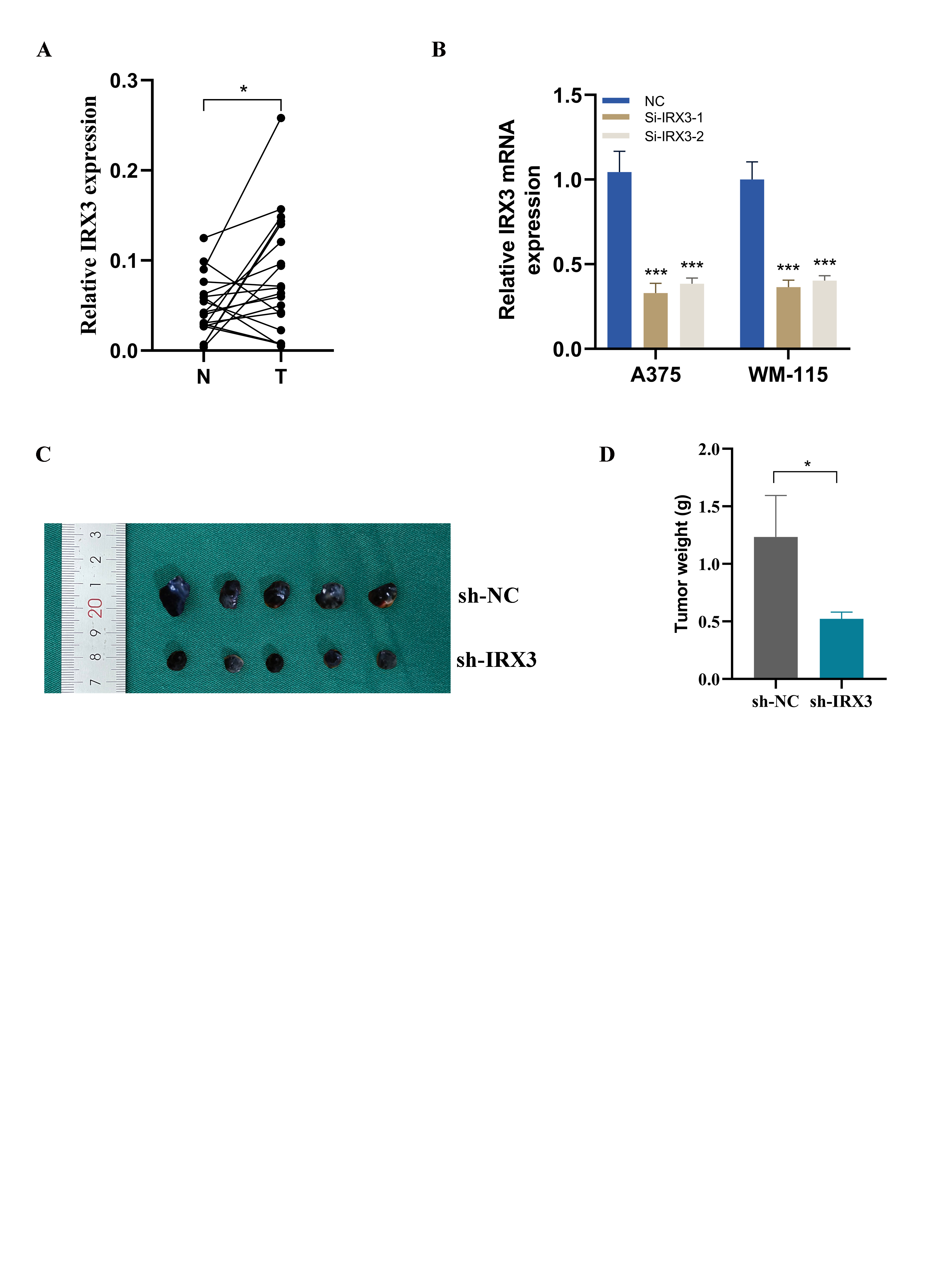

Supplement: Supplementary Figure 3 — IRX3 expression level between melanoma and corresponding normal tissues and IRX3 silencing efficiency. (A) A significant upregulation mRNA level of IRX3 was observed in the melanoma group among 20 pairs of melanoma and corresponding normal tissues. (B) Confirmation of knockdown efficiency through qPCR in A375 and WM-115 cell lines. (C) Photographs of tumors obtained from the different groups of nude mice transfected with sh-NC and sh-IRX3. (D) Knockdown of IRX3 expression significantly inhibited melanoma cancer cell growth in nude mice and tumor weight was significantly reduced in the sh-IRX3 group compared to that in the NC group. [file Image_3.tif]
