# Supplementary material for: Single-cell sequencing analysis related to sphingolipid metabolism guides immunotherapy and prognosis of skin cutaneous melanoma
Source: Front Immunol. 2023 Nov 23;14:1304466. doi: 10.3389/fimmu.2023.1304466 (PMC10701528; doi:10.3389/fimmu.2023.1304466)
Supplement: Supplementary file 4 [file Table_1.docx]

| **Oligonucleotides** | **Nucleotide sequence (5'-3')** |
| --- | --- |
| **siRNA** |  |
| Scramble control | GCUUCGCGCCGUAGUCUUA |
| Si-IRX3-1 | GAGATCGATTTGGAGAACTTA |
| Si-IRX3-2 | AGTGCCTTGGAAGTGGAGAAA |
|  |  |
| **Primer** |  |
| GAPDH | GGCCTCCAAGGAGTAAGACC (forward) |
|  | AGGGGAGATTCAGTGTGGTG (reverse) |
| IRX3 | GCGGCCCCAGAACCATC (forward) |
|  | CCCCCAAAATCAACCGGACA (reverse) |
|  |  |

**Table S1. Oligonucleotides used in research**
